# Lung Function, Dietary Intake, and Weight Status in Children with Persistent Asthma from Low-Income, Urban Communities

**DOI:** 10.3390/nu11122943

**Published:** 2019-12-03

**Authors:** E. Whitney Evans, Daphne Koinis-Mitchell, Sheryl J. Kopel, Elissa Jelalian

**Affiliations:** 1 Weight Control and Diabetes Research Center, The Miriam Hospital, Providence, RI 02903, USA; Elissa_Jelalian@brown.edu; 2 Department of Psychiatry and Human Behavior, Brown University Alpert School of Medicine, Providence, RI 02903, USA; 3 Bradley/Hasbro Children’s Research Center, Brown University Medical School, Providence, RI 02903, USA; daphne_koinis-mitchell@brown.edu (D.K.-M.); SJKopel@lifespan.org (S.J.K.)

**Keywords:** asthma, diet, lung function, obesity, overweight, children

## Abstract

Objective: Asthma and obesity are prevalent chronic childhood diseases that commonly co-occur in youth from low-income, minority backgrounds. Diet is a known risk factor for obesity; however, its role in asthma/obesity comorbidity is not well established. This analysis examined the association between diet and lung function and effect modification by weight status. Methods: Lung function (FEV_1_ % predicted), anthropometric, and dietary data were collected from 95 children, ages 7–9 years old with persistent asthma, from low-income, urban communities in the United States. Associations between lung function, diet and weight status were examined using multivariable linear regression. Results: There were no significant differences in dietary intake between children with persistent asthma with and without obesity; however, >85% of participants did not meet recommendations for fruit, vegetable, and whole grain intake for their age and sex. Only intake of fruit (whole fruit and juice) was significantly associated with FEV_1_ % predicted (−3.36; 95% CI: −6.5 to −0.2). Conclusions: Diet quality was poor in this sample, independent of weight status. More research is needed to understand the relationship between diet, lung function, and weight status, so that interventions can be developed to concurrently address obesity and weight.

## 1. Introduction

Asthma is the most prevalent childhood chronic illness in the US [1,2]. Approximately 13.8% or 10 million American children have asthma, on average; yet, rates are over 20% in urban centers where exposure to environmental allergens and irritants is high [3,4,5]. Moreover, the prevalence of asthma is higher in African-American and Hispanic populations, particularly Puerto Rican children, and asthma morbidity is greater in these groups as compared to non-Hispanic white (NHW) children [6,7,8,9]. Childhood asthma is associated with high health care utilization often involving preventable emergency room visits and hospital admissions, and is responsible for over 13 million missed school days annually [10].

The co-occurrence of childhood asthma and overweight and obesity (OW/OB) is well documented, with close to 50% of youth with asthma categorized as having overweight or obesity [11]. Some studies have found that children with asthma are at greater risk for OW/OB, while others show that having OW/OB may increase risk for the development of asthma [12,13,14]. Regardless of the directionality of the relationship, overlapping genetic, developmental, lung mechanical, immunological, and behavioral factors have all been implicated [15]. The highest co-morbidity occurs in Hispanic and African American children and in those from low-income and/or urban settings [1,2,3,16,17,18,19,20,21,22]. While some risk factors shared by asthma and OW/OB are not modifiable, others, including behaviors such as diet, physical activity, and sedentary time, are modifiable and may lessen the burden of these two conditions, especially among urban minority youth [17,23]. Thus, research is needed to elucidate shared behavioral processes to identify those at greatest risk and to inform both prevention and treatment approaches.

Diet may help to explain one shared pathway between these conditions, as it is a risk factor for both asthma and OW/OB. The relationship between dietary intake, both quantity and quality, and excess weight gain in children is well established [24,25]. Similarly, multiple aspects of diet have been shown to increase or decrease the risk for asthma prevalence and/or symptoms. Cross-sectional data suggest that high daily consumption of sugar-sweetened beverages, snacks, sweets and dairy is associated with increased odds of having asthma [26,27]. Findings from the ISAAC Phase Three study, a cross-sectional study of children living in Latin American countries (The International Study on Asthma and Allergies in Children; ISAAC), suggest that fast food consumption three or more times per week is positively associated with risk for current wheeze, while fruit and vegetable consumption is associated with reduced odds of having asthma and current wheeze [28,29,30]. Finally, dietary pattern analyses suggest that a diet rich in fruit, vegetables, and fish, similar to the Mediterranean diet, is associated with lower asthma risk, better lung function, and lower use of asthma medications (bronchodilators and corticosteroids) among children with asthma [31,32,33,34,35,36].

While there is a growing literature supporting the association between dietary intake and asthma symptoms/lung function, few studies have investigated this relationship in urban, ethnically diverse children with diagnosed persistent asthma. Further, how weight status affects the association between diet and lung function has not been well studied. Therefore, research is needed to clarify whether dietary intake contributes to variations in lung function among urban children with persistent asthma with and without OW/OB. 

The primary objective of the current study was to examine the association between dietary intake and lung function in urban children with persistent asthma. As secondary outcomes, we sought to characterize dietary intake of children with asthma with and without OW/OB, and examine whether the association between diet and lung function (FEV_1_ % predicted) is moderated by weight status. We hypothesized that dietary intake, particularly the consumption of fruit, would be associated with better lung function in this sample of racially and ethnically diverse urban children with persistent asthma, and that OW/OB would modify this relationship, such that the potential benefits of fruits on lung function would not be as strong in children with OW/OB. 

## 2. Methods

Data for this investigation were collected from a larger study (R01HL116254, Koinis Mitchell and Jelalian, PIs), which was designed to examine the association between asthma and participation in physical activity in urban children, ages 7–9 years old with persistent asthma, and multi-level factors that contribute to this relationship. Data included herein were collected between the fall of 2013 and the spring of 2016 as part of clinic visits that occurred as part of this larger observational study, which is described elsewhere [37,38]. All study assessments were administered in English or Spanish by research staff fluent in both languages, and per each participant’s preference. Families were compensated with small cash payments at the end of each research visit. The parent study was approved by the Institutional Review Board at Rhode Island Hospital. 

## 3. Participants

Children were enrolled from one of four large urban school districts in a Northeastern state in the United States of America, through hospital-based, ambulatory pediatric clinics, or from a hospital-based asthma educational program. Eligibility criteria for the study at screening included: (1) an age of between 7 and 9 years old, (2) the child’s legal guardian being willing to participate, (3) the caregiver ethnicity’s being self-identified as Latino (Dominican or Puerto Rican), non-Latino white, or black/African American, (4) the incidence of physician-diagnosed asthma or breathing problems in the last 12 months, and (5) the child had attended public school in 1 of 4 of the largest urban school districts in the state in which the study took place. In addition, each child was required to meet the diagnosis of persistent asthma at study screening, either by current prescription of an asthma controller medication, and/or recurrent daytime symptoms, nighttime symptoms, activity limitation, rescue medication use, or ≥2 oral steroid bursts during the previous 12 months. During the study, confirmation of asthma diagnosis and persistent severity status was made by a study clinician. Exclusion criteria were moderate-to-severe cognitive impairment as determined by school placement, use of stimulant medication for ADHD, another pulmonary or chronic health condition, or a diagnosed sleep disorder (e.g., restless leg syndrome, chronic insomnia). 

## 4. Measures

### 4.1. Demographic Information 

Primary caregivers provided key demographic information (see Table 1). Poverty status was determined by dividing the family’s annual income by the Federal per capita poverty threshold for a family of that size in the year of the family’s participation in the study [39,40]. 

### 4.2. Asthma Diagnosis/Persistent Severity Status 

Confirmation of asthma and classification of severity were made by a study clinician at the clinic visit using standard NHLBI EPR-3 guidelines [2]. Current asthma medication use was also confirmed, and caregivers provided ratings indicating how often their child missed doses of daily controller medications on a scale from 1 (misses doses all of the time) to 5 (never misses doses). Lung function was assessed during the clinic visit using Koko incentive spirometer (nSpireHealth, Longmont, CO, USA) before and after short acting *β*-agonist administration [41]. Koko spirometer was used to obtain measurements of forced vital capacity (FVC), forced expiratory flow (FEV_1_ % predicted), and forced expiratory flow, with 25%–75% vital capacity (FEV_25-75%_). 

### 4.3. Asthma Control 

Parents and children completed the Asthma Control Test (ACT), a well-validated questionnaire commonly used in the classification of asthma severity. In accordance with previously used procedures, scores on the ACT were dichotomized, with a total cutoff score below 19 indicating suboptimal asthma control, and ≥19 indicating well-controlled asthma [42,43,44,45].

### 4.4. Anthropometrics 

Trained research assistants obtained each participant’s weight and height during a clinic visit. Weight was measured in street clothes, without shoes, using a digital scale to the nearest 0.1 kilogram. Height was measured using a portable stadiometer to the nearest millimeter. BMI-for-age percentile and BMI-for-age z-scores (BMIz) were calculated using the Centers for Disease Control and Prevention (CDC) standards [46]. Children with a BMI < 85^th^ percentile for age and sex were categorized as having normal weight, those with BMI ≥ 85^th^ percentile and < 95^th^ percentile for age and sex were categorized as being overweight, and those with a BMI > 95^th^ percentile for age and sex where categorized as being obese [47]. Children with overweight and those with obesity were examined as one group given the similarities in comorbidity risk in both groups and FEV1 % predicted scores (overweight: 86.9 ± 10.8 vs. obesity: 84.2 ± 14.4; *p* = 0.47) [48].

### 4.5. Dietary Intake 

Children’s usual, relative dietary intake was measured using the Block Kids Food Screener (BKFS; NutritionQuest, Berkley, CA, USA). The BKFS, which asks about intake during the past week, is designed to measure dietary intake by food groups and focuses on the intake of fruits, juices, vegetables, breakfast cereal, protein sources, dairy, soda, sweets, and high-fat snacks. The BKFS has been validated using 24–hour diet recalls in middle and high school students [48], and provides an estimate of total energy intake. 

### 4.6. Statistical Analysis

All statistical analyses were conducted using SAS 9.4 (2014; SAS Institute, Inc., Cary, NC, USA), at the two-sided 0.05 level of significance. General descriptive statistics were generated for demographics and anthropometrics and differences by weight status group (healthy weight: BMI < 85^th^ percentile for age and sex vs. those with OW/OB: BMI ≥85^th^ percentile for age and sex) were assessed using Student’s T- and Chi-square tests as appropriate. For participants with complete anthropometric and dietary data and missing lung function data (*n* = 5), FEV_1_ % predicted values were determined using matching. Specifically, participants with missing FEV_1_ % predicted values were matched by sex, age (years), race/ethnicity and baseline asthma severity with participants with complete data. A mean FEV_1_ % predicted value was determined from all matched cases and assigned to the participant with missing data. Chi-square tests were used to determine the frequency of participants meeting dietary recommendations for their age and sex. Multivariable linear regression was used to estimate the relationships of lung function (FEV_1_ % predicted) with adiposity measures (BMI z-score and waist circumference) and diet (cup equivalents of fruits, vegetables, whole grains, meat, poultry and fish, and added sugars). Each dietary variable was examined in a separate model, and the assumptions of linear regression were met by the data. To determine if the relationship of diet and lung function differed in children with normal weight vs. OW/OB, we tested the significance of an interaction term for weight status and dietary intake in the multivariable linear models. A *priori*, all multivariable linear regression models controlled for sex, race/ethnicity, poverty status, and estimated total energy intake. 

## 5. Results

A total of 147 children enrolled in the larger study; however, the analytic sample included 90 children who had complete lung function, anthropometric, and Block kid’s dietary screener data and five children with complete anthropometric and dietary data as well as imputed lung function data. When grouped by weight status, 51.6% (*n* = 49) of the sample had a weight consistent with OW/OB. As shown in Table 1, children with normal weight and those with OW/OB did not differ based on their race/ethnicity (19.6 vs. 18.4 NLW) or poverty status. While not statistically significantly different, there were more children with OW/OB who had severe persistent asthma as compared to children with normal weight (22.4% vs. 13.1%, respectively); however, the two weight status groups did not differ with respect to lung function or asthma control. 

As shown in Table 2, we compared the reported food group intake (as measured in cup or ounce equivalents) in children with and without OW/OB to dietary recommendations for their age and sex. No differences were found between groups; however, independent of weight status, more than 85% of participants did not meet recommendations for fruit and vegetable intake for their age and sex. Similarly, 100% of participants in the sample did not meet the recommendation for whole grain intake. More than 40% of participants exceeded the recommendation to limit added sugar to six teaspoons per day for females and nine teaspoons for males. Finally, despite the strong evidence recommending avoidance of sugar-sweetened beverages, nearly half the sample consumed them daily. 

To understand how diet may relate to lung function and obesity co-morbidity, we examined the linear relationship between food group intake and lung function, as measured by FEV_1_ % predicted. As shown in Table 3, each additional cup equivalent of fruit intake (whole fruit and juice) was associated to −3.36% of FEV_1_ % predicted (*p* = 0.04) after controlling for sex, race/ethnicity, poverty status and estimated total energy intake. In separate models, the associations of vegetables, whole grains, dairy, meat, poultry and fish, added sugars, fiber and sodium were not statistically significantly related to lung function (FEV_1_ % predicted). Moreover, weight status did not modify the association of any food group with lung function (*p*’s > 0.05).

## 6. Discussion

While the shared mechanisms underlying co-occurring childhood asthma and obesity are not well understood, it is likely that overlapping behavioral risk factors, including diet, play a role. In this carefully evaluated sample of 95 racially and ethnically diverse children from low-income urban communities and with persistent asthma, we found that children consumed an overall poor-quality diet. Moreover, we found that dietary intake, in this sample, was not associated with lung function, as measured by FEV_1_ % predicted.

Another important finding from this study was that among youth with persistent asthma from low-income and diverse racial and ethnic backgrounds, the prevalence of overweight or obesity was more than 50 percent. This is substantially higher than national prevalence data, which suggests that 33% of American youth have OW/OB [49]. The observed prevalence is similar to other samples, as for a cohort of multiethnic children from Southern California, researchers found that 46.5% of the surveyed youth had OW/OB [50]. This finding supports the co-occurrence of asthma and obesity and emphasizes the need for research in high-risk populations.

Based on evidence that consuming a diet low in fruits and vegetables, high in added sugars, energy-dense snacks and sweets and fast foods, and low in omega-3 fatty acids is independently associated with a higher prevalence of asthma and obesity, we anticipated that children with asthma and OW/OB would be less likely to meet the recommendations for daily fruit, vegetable, and whole grain intake and more likely to consume more added sugars and sugar-sweetened beverages that is recommended [26,36,50,51,52]. However, findings from this sample do not support differences in food group intake by weight status. Instead, we found that the majority of participants, independent of weight status, did not meet daily recommendations for fruits, vegetable or whole grain intake. Moreover, nearly half of participants reported consuming at least one sugar-sweetened beverage per day, despite consistent public health messages to avoid sugar-sweetened beverages based on their strong association with obesity and other co-morbidities [53,54]. These results contribute to the literature as previous work in this area has not considered how dietary intake of children with asthma and obesity compares to national recommendations. Our findings are also consistent with evidence that children from low-income families often consume low quality diets due to barriers of cost and accessibility of nutrient-dense foods [55,56]. Thus, they emphasize that intervening on poor diet, and the poverty that often causes it, is a necessary component for interventions aimed at lowering the burden of both asthma and obesity in similar populations.

The primary objective of this analysis was to examine the associations of food group intake with lung function and to determine whether associations differed by weight status. In this sample, intake of vegetables, whole grains, meat, poultry and fish, or added sugars were not related to lung function measurements. Consumption of fruit was associated with a lower FEV_1_ % predicted. This finding is inconsistent with the literature, which suggests that fruit intake is associated with reduced wheeze and higher FEV_1_ % predicted in children with asthma [35,57]. However, we attribute this unexpected finding to the Block Kid’s Food Screener, which does not differentiate between whole fruit consumption and fruit juice intake. Nationally representative data suggest that children who consume five or more beverages high in fructose per week, which include sugar-sweetened beverages, apple juice, and fruit drinks, have 5.29 times the odds of having asthma as compared to those who consumed one or fewer [58]. Thus, participants in this sample may consume more fruit juice than whole fruit, which led to a spurious association between overall fruit intake (whole and juice) and reduced FEV_1_ % predicted. Finally, we did not find that OW/OB modified the relationships of any food group intake with lung function (FEV_1_ % predicted). While unexpected, we did not observe any dietary differences in participants with persistent asthma with and without OW/OB, such that this may need to be examined in a sample with more variety in dietary intake.

The primary strength of this study is that it was carried out in a sample of racially and ethnically diverse children with persistent asthma who were from low-income, urban communities. Another strength of this paper is that we examined the relationship of asthma, weight status and diet, which has not been done within this population. This study is not without limitations, however. First, only 90 children from the parent study had complete lung function, anthropometric and dietary data. Given that these children were from one specific geographic region in the US, the generalizability of our findings is limited. Second, the Block Kid’s Food Screener may not have included foods that routinely contribute to participant’s usual diet in this sample of youth. Finally, it is possible that we have not included all relevant confounders in our analyses, as we did not measure exposure to environmental tobacco smoke or other household triggers that may affect lung function and asthma control in urban children. Each of these limitations along with the potential for regression toward the mean, increase the risk of a type two error.

Findings from this study suggest that in a sample of racially/ethnically diverse youth with persistent asthma, adherence to national dietary recommendations was low, independent of weight status. Intervening on diet may yield meaningful health benefits in similar populations. Moreover, while the majority of associations between food group intake and lung function were not statistically significant in this sample, it is possible that interventions designed to improve diet and reduce inflammation may lead to concurrent weight loss and improved lung function. Further research is warranted with a more precise measure of diet and biomarkers of inflammation, so as to elucidate potential mechanisms of shared behavioral risk factors for the two common childhood diseases.

## Figures and Tables

**Table 1:** Demographics and measures of lung function in children with asthma who have a normal weight vs. overweight (OW)/obesity (OB).

	Normal Weight (*n* = 46)	OW/OB (*n* = 49)	*p*-Value
Age (years ± SD)	8.1 + 0.9	8.1 + 0.8	0.98
% Female	45.6%	44.9%	0.94
Race/Ethnicity			
Latino	52.2%	53.0%	0.99
Non-Hispanic Black	28.3%	28.6%	
Non-Hispanic White	19.6%	18.4%	
SES (Lives below the Poverty Line)	68.9%	67.4%	0.88
Baseline Asthma Severity			
Mild Intermittent	6.5%	2.0%	0.50
Mild Persistent	50.0%	46.9%	
Moderate Persistent	30.4%	28.6%	
Severe Persistent	13.1%	22.4%	
Forced Expiratory Volume (FEV)	85.0 ± 13.4	85.5 ± 12.7	0.72
Forced Vital Capacity	85.6 ± 17.3	84.9 ± 19.9	0.85
FEV below 80% predicted	30.4%	34.8%	0.65
Asthma Control Test (<19 = poorly controlled)	31.1%	25.5%	0.55

**Table 2:** Proportion of youth with asthma and normal weight or OW/OB who DO NOT meet dietary recommendations for their age and sex ^1^.

	Normal Weight (*n* = 46)	OW/OB (*n* = 49)	*p*-Value
Nutrients and Food Groups to Increase (Adequacy)			
Fruit and vegetable intake ^2^	87.0%	85.7%	0.86
Whole grain intake ^3^	100%	100%	--
Fiber (g) ^4^	100%	98.0%	0.33
Nutrients and Food Groups to Decrease (Moderation)			
Added sugar intake ^5^	41.3%	46.9%	0.58
Sugar-sweetened beverages intake ^6^	47.8%	55.1%	0.47
Sodium (mg) ^7^	30.4%	30.6%	0.98

^1^ Dietary recommendations identified from the Dietary Guidelines for Americans, 2010 for age and sex specific groups. ^2^ Fruit & Vegetable intake recommendations: Ages 4–8 years: 2.5 cups/day for males & females; Ages 9–13 years: 4 cups/day for males, 3.5 cups/day for females.^3^ Whole Grain intake recommendations: Ages 4–8 years: 2 to 4 oz/day for females, 2.5 to 5 oz/day for males; Ages 9–13 years: 3 to 5 oz/day for females, 3 to 6 oz/day for males. ^4^ Fiber recommendations: Ages 4–8 years: 25 grams/day; Ages 9–13 years: 26 grams/day for females, 31 grams/day for males. ^5^ Added sugars: Females: <6 tsp, Males: <9 tsp. ^6^ Sugar-sweetened Beverages: 0 for all ages, sexes. ^7^ Sodium: Ages 6–18 years: <2300 mg per day.

**Table 3:** Linear relationship between diet and lung function, as measured by FEV1 % predicted, in children with persistent asthma from a low-income, urban setting.

	Regression Coefficient (95% CI) ^2^	*p*-Value	R^2^
Fruit, CE ^1^	−3.36 (−6.5 to −0.2)	0.04	0.11
Vegetables (no potato), CE	9.97 (−0.31 to 20.3)	0.06	0.10
Whole grains, ounces	−3.96 (−11.1 to 3.2)	0.27	0.07
Meat, fish, and poultry, ounces	1.76 (−0.4 to 3.9)	0.1	0.09
Dairy	1.84 (−1.8 to 5.5)	0.32	0.07
Added sugar (teaspoons)	−0.60 (−1.4 to 0.2)	0.12	0.08
Fiber (grams)	0.06 (−0.94 to 0.81)	0.88	0.05
Sodium (milligrams)	0.006 (−0.002 to 0.015)	0.15	0.08

^1^ Cup Equivalents (CE). ^2^ Separate regression models controlling for sex, race/ethnicity, poverty and estimated daily total energy intake.
